# Effects of Dietary Arginine, Ornithine, and Zeolite Supplementation on Uremic Toxins in Cats

**DOI:** 10.3390/toxins10050206

**Published:** 2018-05-18

**Authors:** Nadine Paßlack, Jürgen Zentek

**Affiliations:** Institute of Animal Nutrition, Department of Veterinary Medicine, Freie Universität Berlin, Königin-Luise-Straße 49, 14195 Berlin, Germany; Juergen.Zentek@fu-berlin.de

**Keywords:** urea, blood, uremic toxins, liver, kidneys

## Abstract

To test if arginine and ornithine, both components of the Krebs-Henseleit cycle, or zeolite, a potential ammonium absorber, can modulate the excretion of harmful bacterial metabolites, intestinal microbial protein fermentation was stimulated by feeding a high-protein (60.3%) diet as a single daily meal to 10 adult cats. The diet was supplemented without or with arginine (+50, 75, 100% compared to arginine in the basal diet), ornithine (+100, 150, 200% compared to arginine in the basal diet), or zeolite (0.125, 0.25, 0.375 g/kg body weight/day). The cats received each diet for 11 days. Urine, feces, and blood were collected during the last 4 days. Arginine and ornithine enhanced the postprandial increase of blood urea, but renal urea excretion was not increased. Zeolite decreased renal ammonium excretion and fecal biogenic amines. The data indicate an increased detoxification rate of ammonia by arginine and ornithine supplementation. However, as urea was not increasingly excreted, detrimental effects on renal function cannot be excluded. Zeolite had beneficial effects on the intestinal nitrogen metabolism, which should be further evaluated in diseased cats. Clinical studies should investigate whether dietary arginine and ornithine might improve hepatic ammonia detoxification or could be detrimental for renal function.

## 1. Introduction

Undigested protein undergoes microbial fermentation in the large intestine [1]. The resulting metabolites include ammonia, biogenic amines, phenols, indoles, and branched-chain fatty acids [2], which exert varying effects in the organism [2,3]. So-called uremic toxins can be considered as a group of waste products originating predominantly from bacterial protein breakdown [4]. As a consequence of an impaired excretory capacity, uremic toxins are retained in the blood of individuals suffering from chronic kidney disease [5]. This accumulation of waste molecules can promote the progression of chronic kidney disease, as well as contribute to inflammation in the organism and cardiovascular disease [5,6,7]. Examples of uremic toxins are urea, p-cresyl sulfate, indoxyl sulfate, and hippuric acid [4]. The European Uremic Toxin Work Group has currently published 130 different uremic solutes in their database (http://www.uremic-toxins.org/DataBase.html).

Dietary strategies to reduce uremic toxins in the blood of human individuals especially focus on a modulation of the intestinal microbiota by the use of pre- and probiotics, and on the dietary supplementation of intestinal adsorbents [8,9]. In cats, however, little data are available on dietary interventions to reduce uremic toxins in the blood. In one study, a combination of calcium carbonate and chitosan lowered (*p* < 0.05) urea and phosphate concentrations in the plasma of cats [10]. 

In the present study, new dietary strategies to modulate the excretion of microbial metabolites arising from intestinal protein fermentation were evaluated in cats. Although cats were the target species for this investigation, the results might also be highly interesting for human individuals. Both cats and humans show a high prevalence for chronic kidney disease [11], and the potential of dietary supplements to modulate the excretion of uremic toxins is of particular relevance for both species.

On one side, arginine and ornithine, both components of the Krebs-Henseleit cycle [12], were supplemented to a diet. It was hypothesized that the detoxification rate of ammonia to urea could be improved by these supplements. In human medicine, dietary arginine supplementation is used for the treatment of defects of the urea cycle [13]. Besides a promotion of hepatic ammonia detoxification, a restoration of creatine and nitric oxide production and increased protein synthesis are described as beneficial effects of arginine supplementation in diseased individuals [13]. However, to our best knowledge, no data are available on the efficacy of arginine or ornithine to modulate ammonia detoxification in cats.

Besides arginine and ornithine, the dietary supplementation of zeolite was evaluated in the present study. Zeolite has been demonstrated to bind ammonium in vitro [14]; however, no data are available on its effects in cats.

To test the effects of dietary arginine, ornithine, and zeolite, enhanced blood urea concentrations of the cats were experimentally induced. The cats showed neither impaired kidney nor liver function prior to the conduction of the study. However, elevated blood urea concentrations were induced by feeding a high-protein diet in combination with only a single meal per day. It was assumed that the high protein intake would enhance the bacterial fermentation of undigested protein in the large intestine, allowing us to evaluate the potential effects of the dietary supplements to modulate the excretion of selected end products of the microbial protein metabolism.

## 2. Results

### 2.1. Blood Parameters

The dietary supplementation of arginine, ornithine, and zeolite did not affect the fasting blood urea concentrations of the cats (*p* > 0.05) (Table 1). However, ornithine increased postprandial blood urea concentrations from 12.0 mmol/L (basal diet) to 13.5 mmol/L (Orn3; linear contrast: *p* = 0.003). In addition, the postprandial urea increase (%) was calculated in order to evaluate the relationship between the fasting and postprandial blood urea concentrations. Both arginine and ornithine supplementation enhanced the postprandial urea increase (%) in the blood of the cats (linear contrast for arginine: *p* = 0.007; linear contrast for ornithine: *p* = 0.027), whereas no effect was observed following dietary zeolite supplementation. Fasting and postprandial creatinine, phosphate, and magnesium (Mg) concentrations in the blood of the cats were neither affected by the dietary supplementation of arginine nor ornithine nor zeolite (*p* > 0.05).

### 2.2. Body Weight, Feed Intake, Urine Volume, and pH, Amount and Dry Matter (DM) of the Feces

Dietary arginine, ornithine, and zeolite supplementation had no effect on body weight, feed intake, or urine pH of the cats (Table 2). The urine volume decreased from 28.3 mL/kg body weight/day (basal diet) to 25.4 mL/kg body weight/day when zeolite was added to the diet (Ze3; linear contrast: *p* = 0.029). Dietary ornithine supplementation decreased the amount of feces from 7.83 g/kg body weight/day (basal diet) to 7.58 g/kg body weight/day (Orn3; linear contrast: *p* = 0.026). Supplementation of zeolite to the diet first decreased DM content of the feces (Ze1), but increased fecal DM at higher supplementation doses (Ze2–Ze3; linear contrast for zeolite: *p* = 0.039; quadratic contrast for zeolite: *p* = 0.029).

### 2.3. End Products of the Protein Metabolism

The detection of selected end products of the protein metabolism in the urine and feces of the cats revealed variations depending on the dietary supplementation of arginine, ornithine, and zeolite (Table 3 and Table 4). Arginine decreased renal urea excretion (linear contrast: *p* = 0.039; quadratic contrast: *p* = 0.047), but increased urinary creatinine (linear contrast: *p* = 0.005) and hippuric acid concentration (linear contrast: *p* = 0.003). Renal ammonia excretion decreased from 31.9 mg/kg body weight/day (basal diet) to 25.9 mg/kg body weight/day when zeolite was added to the diet (linear contrast: *p* = 0.038). A quadratic effect was observed for renal sulfate excretion (arginine supplementation; *p* = 0.043) and urinary hippuric acid concentration (ornithine supplementation; *p* = 0.038).

In the feces of the cats, dietary ornithine supplementation resulted in a decrease of ammonia (linear contrast: *p* = 0.014) and 4-ethylphenol (linear contrast: *p* = 0.026) concentrations, whereas zeolite decreased the concentrations of putrescine (linear contrast: *p* = 0.002) and total biogenic amines (linear contrast: *p* = 0.006). Fecal crude protein concentrations were linearly decreased from 498 mg/g DM to 454 mg/g DM by dietary zeolite supplementation (*p* = 0.001), while only a quadratic effect was observed following dietary arginine supplementation (*p* = 0.025) (Table 5). Dietary ornithine first decreased (Orn1–2), but subsequently increased (Orn3) the fecal crude protein excretion of the cats (linear contrast: *p* = 0.026; quadratic contrast: *p* = 0.049), and generally increased the apparent digestibility of crude protein (linear contrast: *p* = 0.035).

## 3. Discussion

It was the hypothesis of the present study that the dietary supplementation of arginine, ornithine, and zeolite could modulate the excretion of selected end products of the protein metabolism in cats. Different underlying mechanisms were assumed, on one side an increased detoxification of ammonia to urea by arginine and ornithine, both components of the Krebs-Henseleit cycle [12], and an associated enhanced renal urea excretion. However, the present study demonstrated an enhanced postprandial increase of urea in the blood of the cats when arginine and ornithine were supplemented to the diet, but a reduced (arginine supplementation) or unaffected (ornithine supplementation) renal urea excretion. The urea concentrations in the urine of the cats were unaffected among all treatment groups. It can be assumed that the high protein intake of the cats, resulting from a high dietary protein level in combination with a high food intake per meal, increased the microbial fermentation of undigested protein in the large intestine and therefore also enhanced the microbial production and intestinal absorption of ammonia. Thus, a higher detoxification rate of ammonia in the Krebs-Henseleit cycle might explain the increased postprandial urea concentrations (mmol/L) in the blood of the cats, which were above the reference value among all groups. However, the enhanced postprandial increase (%) of blood urea concentration observed following arginine (*p* = 0.007) and ornithine supplementation (*p* = 0.027) might indicate a promotion of the Krebs-Henseleit cycle by these dietary supplements. In human medicine, dietary arginine supplementation is considered for patients with defects of the urea cycle in order to restore the detoxification of ammonia [13]. Based on the present results, an effect of dietary arginine on the detoxification rate of the Krebs-Henseleit cycle might also be assumed. However, as the dietary supplements also increased the nitrogen intake of the cats, although only to a minor degree, this aspect could also have contributed to the enhanced postprandial increase of blood urea. Nevertheless, the increased concentrations of urea were accumulated in the blood of the cats, but not increasingly excreted by the kidneys. This might indicate that the excretory mechanisms were overstressed or that the renal function might have been negatively impacted. In fact, it has been reported that excessive doses of arginine can induce renal failure in rats [15]. This effect was explained by the production of uremic toxins, such as nitric oxide, methylguanidine, or creatinine, from excessive amounts of dietary arginine, and their potential damaging effects on renal function [15]. In the present study, only the postprandial blood urea concentrations were elevated, not the fasting blood urea concentrations. This suggests a temporary effect on the kidneys’ excretory function. Nevertheless, the elevated postprandial increase (%) of blood urea when arginine and ornithine were supplemented to the diets should be further evaluated in diseased cats, particularly in animals with impaired liver and kidney function, in order to clarify whether dietary arginine and ornithine would be beneficial with regard to an improvement of the hepatic ammonia detoxification, or, on the contrary, whether such supplementation may also be detrimental for renal function. In this context, it should also be considered that the cats of the present study received a high-protein diet in order to stimulate the microbial protein fermentation in the large intestine and, as a consequence, to experimentally increase the blood urea concentration. However, this experimental approach also implied a high arginine intake by the basal diet. Thus, due to the high baseline intake, the effects of an additional supplementation might have been less pronounced. In practice, a low-protein diet would be used for the dietary treatment of cats with an impaired liver or kidney function. It can be hypothesized that the effects of dietary arginine or ornithine supplementation would become more obvious at generally low protein (and therefore amino acid) intakes. On the other hand, it might also be possible that the amounts of the supplements have to be relatively high in diseased individuals in order to achieve comparable intakes and the associated effects, as seen in the present study. At this stage, it can be concluded that arginine and ornithine supplementation had an impact on blood urea concentrations in cats, but that further clinical studies are required to provide specific dietary recommendations.

The dietary supplementation of zeolite was evaluated with regard to its potential to absorb ammonia in the intestine, which would lower the need for ammonia detoxification in the liver and subsequently lower the production of urea. Zeolite belongs to the group of the aluminosilicates [16] and has been demonstrated to show high absorption rates for ammonium in vitro [14]. In the present study, the renal ammonium excretion of the cats was reduced when zeolite was added to the diet (*p* = 0.038). Nevertheless, neither fecal ammonium concentrations nor urea concentrations in the blood of the cats were affected by the dietary zeolite supplementation. Thus, it could not be clearly demonstrated that zeolite reduced the intestinal absorption of ammonia and its detoxification to urea. On the other hand, the reduced renal ammonium excretion and the observed lower fecal concentrations of biogenic amines, which are bacterial metabolites of the amino acid decarboxylation in the intestine [3], indicate a beneficial impact of zeolite on the intestinal nitrogen availability. It should be considered that the present study induced elevated blood urea concentrations by feeding a high-protein diet; the cats did not suffer from chronic kidney disease. As mentioned above, a typical renal diet has markedly lower protein concentrations than that employed in the present study, which may result in lower ammonium concentrations in the intestine. Future studies should evaluate the ammonium-binding capacity and relevance of zeolite when feeding a low-protein diet, especially in cats with impaired liver or kidney function. The present data on the reduced renal ammonium excretion and fecal biogenic amines concentrations can be considered as a first indicator for a potential impact of dietary zeolite on nitrogen metabolism in cats.

The present study further evaluated the effects of zeolite on the phosphate and Mg concentrations in the blood of cats. In dairy cows, zeolite A is a calcium binder used as a dietary strategy to prevent hypocalcemia around calving [17,18]. However, it has also been demonstrated that high doses of zeolite A can reduce P [19,20] and Mg concentrations [17,19] in the blood of dairy cows. In the present study, however, the blood levels of phosphate and Mg did not differ among the groups and were always within the reference range.

In conclusion, the present study experimentally induced enhanced blood urea concentrations in cats by feeding a high-protein diet and only a single meal per day. The dietary supplementation of arginine and ornithine, both components of the Krebs-Henseleit cycle, enhanced the postprandial increase (%) of blood urea concentrations in cats. On one side, this effect might indicate a promotion of the detoxification rate of ammonia in the Krebs-Henseleit cycle, which might be of practical interest for cats suffering from liver diseases. However, as urea was accumulated in the blood, but not increasingly excreted by the kidneys, detrimental effects on the kidney function, as previously assumed for excessive arginine in rats [15], cannot be excluded. Thus, future studies should evaluate the relevance of arginine and ornithine supplementation in cats with impaired liver and kidney function.

Dietary zeolite reduced the renal ammonium excretion and the concentrations of biogenic amines in the feces of the cats. This might indicate an impact of zeolite on the intestinal nitrogen metabolism; however, further studies are required to investigate the relevance of these results especially in diseased cats.

## 4. Materials and Methods

### 4.1. Study Design

The present study was approved by the Animal Welfare Committee (Landesamt für Gesundheit und Soziales, Berlin, Germany, G 0120/15). Ten healthy adult cats (four ♀ intact, six ♂ neutered; 7.37 ± 2.00 years at the beginning of the study) received a complete canned diet (Table 6) once a day. The analyzed crude protein concentration of this basal diet was 60.3% in DM. The high dietary protein level in combination with the provision of only one single meal per day was assumed to enhance the influx of undigested protein in the large intestine of the cats, resulting in an increased microbial protein fermentation. To test the hypothesis that arginine, ornithine, or zeolite can modify the excretion of selected end products of protein metabolism, 10 dietary treatments were evaluated in the present study: The basal diet without supplement (W/O); supplementation of arginine to the basal diet in the following amounts (Arg1–3): +50%, +75%, and +100% compared to the arginine provision by the basal diet; ornithine supplementation to the basal diet (Orn1–3): +100%, +150%, and +200% compared to the arginine provision by the basal diet; zeolite supplementation to the basal diet (Ze1–3): 0.125, 0.25, and 0.375 g/kg body weight/day. Each cat received each diet for 11 days, using a randomized cross-over design. Each dietary treatment was followed by a 7-day washout period, where only the basal diet without any supplement was fed. Purified water was offered ad libitum throughout the study.

The cats were housed in the facilities of the Institute of Animal Nutrition of the Freie Universität Berlin under constant temperature (21 °C) and light (12 h light, 12 h darkness) conditions. For the first 7 days of each treatment period and for the washout periods, the cats were housed in groups. For the last 4 days of each treatment period, the cats were individually housed in metabolic cages to collect urine and feces (collection period). Each metabolic cage contained a purpose-built cat litter box with a connected urine collection container for the separation of urine and feces. The urine collection containers were provided with three drops of chlorhexidine-digluconate each in order to prevent bacterial growth in the urine of the cats. The urine was collected twice a day (7.00 h and 12.00 h). At the end of each treatment period, fasting and postprandial blood (7 h after feeding) was collected by venipuncture of the vena cephalica antebrachii.

### 4.2. Blood Analysis

For blood collection, serum and lithium heparin tubes were used. The blood samples were stored at room temperature for 1 h and subsequently centrifuged at 4 °C and 1811× *g* for 10 min (Heraeus Megafuge 1.0R, Thermo Scientific, Karlsruhe, Germany). The plasma concentrations of urea and creatinine as well as the serum phosphate and Mg concentrations were measured using an automatic method (Konelab 60 i Thermo Fisher Scientific).

### 4.3. Nutrient Analysis of the Diets

The crude nutrient, mineral, and amino acid concentrations in the basal diet were analyzed as described elsewhere [21,22,23]. The results of the nutrient analyses are presented in Table 6 and Table 7.

### 4.4. Urine and Feces Analysis

The urine pH was measured daily in the morning using a Seven-Multi pH meter (Mettler-Toledo GmbH, Schwerzenbach, Switzerland). Urinary anion and cation concentrations as well as fecal crude protein concentrations were determined as described elsewhere [21,22]. Fecal biogenic amines were measured according to Paßlack et al. [24].

For the determination of phenols and indoles, 2 g feces were mixed with 1 mL internal standard (5-methylindole + methanol; 1:1) and 4 mL methanol, vortexed, and incubated for 1 h at 4 °C. All samples were prepared in duplicate. During the incubation, the samples were vortexed every 15 min. Subsequently, the samples were centrifuged at 29,000× *g* for 15 min at 4 °C. The supernatant was mixed with 5 mL methanol. All samples were vortexed and incubated for 1 h at 4 °C. During the incubation, the samples were vortexed every 15 min. After the incubation, the samples were centrifuged at 29,000× *g* for 20 min at 4 °C. The supernatants of the duplicates were combined, mixed, and stored at −20 °C overnight. The next day, the samples were thawed, and an aliquot was microfuged at 21,000× *g* for 10 min at 4 °C. The supernatant was analyzed for phenols and indoles using a gas chromatograph (Model 6890N, Agilent Technologies, Santa Clara, CA, USA). The gas chromatograph contained an HP-88 column (length 60 m, internal diameter 250 µm, with a film thickness of 0.2 µm). The initial temperature of the oven was 130 °C, of the injector 230 °C, and of the flame ionization detector 250 °C. The carrier gas was hydrogen gas, produced by a gas generator (Parker ChromGas, Parker Hannifin Corporation, Lakeville, MN, USA). The flow rate was 0.7 mL/min.

### 4.5. Statistics

Data were analyzed using IBM SPSS Statistics 22 (SPSS Inc., Chicago, IL, USA, 2013). Linear and quadratic polynomial contrasts were calculated separately for the arginine, ornithine, and zeolite treatments (General Linear Model repeated measures, within-subject factor: arginine/ornithine/zeolite, number of levels: four (basal diet (W/O) plus three doses of arginine/ornithine/zeolite). The data are presented as means and pooled standard error of means (SEM). The level of significance was *p* < 0.05. 

## Figures and Tables

**Table 1:** Blood urea (mmol/L), creatinine (µmol/L), phosphate (mmol/L), and magnesium (mmol/L) concentrations ^1^, as well as postprandial blood urea increase (%) in cats fed a diet without or with arginine, ornithine, or zeolite supplementation ^2^. *n* = 10/treatment, mean and pooled SEM.

	W/O	Arginine			Ornithine			Zeolite			SEM	Polynomial Contrasts (*P* value)					
												Arginine		Ornithine		Zeolite	
		Arg1	Arg2	Arg3	Orn1	Orn2	Orn3	Ze1	Ze2	Ze3		Lin.	Quadr.	Lin.	Quadr.	Lin.	Quadr.
Urea (fasting)	10.4	10.7	9.92	10.2	9.63	10.5	10.4	10.3	10.1	10.1	0.17	0.299	0.697	0.576	0.470	0.576	0.937
Urea (postprandial)	12.0	12.9	12.8	12.9	12.3	12.9	13.5	12.4	12.1	12.2	0.19	0.089	0.170	**0.003**	0.674	0.958	0.646
Postprandial urea increase	17.5	21.3	29.2	28.1	30.1	24.1	31.1	21.0	19.2	20.1	1.22	**0.007**	0.091	**0.027**	0.397	0.663	0.682
Creatinine (fasting)	137	147	140	143	132	131	133	140	140	135	2.58	0.538	0.442	0.447	0.399	0.701	0.233
Creatinine (postprandial)	116	117	116	113	111	110	112	107	118	118	1.94	0.430	0.304	0.335	0.174	0.384	0.410
Phosphate (fasting)	1.37	1.35	1.28	1.40	1.38	1.31	1.41	1.32	1.39	1.39	0.02	0.922	0.173	0.879	0.264	0.550	0.656
Phosphate (postprandial)	1.61	1.51	1.60	1.51	1.58	1.51	1.54	1.48	1.55	1.62	0.02	0.339	0.872	0.288	0.640	0.748	0.302
Magnesium (fasting)	1.08	1.03	0.99	1.05	1.04	0.99	1.05	1.03	1.06	1.06	0.01	0.496	0.317	0.544	0.240	0.827	0.583
Magnesium (postprandial)	1.22	1.18	1.16	1.16	1.30	1.14	1.22	1.15	1.13	1.13	0.02	0.276	0.697	0.500	0.981	0.154	0.626

^1^ Reference values (Clinic for Small Animals, Freie Universität Berlin): urea (fasting): 5.0–11.3 mmol/L; creatinine (fasting): 53–160 µmol/L; phosphate (fasting): 0.8–1.9 mmol/L; magnesium (fasting): 0.6–1.3 mmol/L. ^2^ Basal diet supplemented without (W/O) or with arginine (Arg1–3: +50%, +75%, and +100% compared to the arginine provision by the basal diet), ornithine (Orn1–3: +100%, +150%, and +200% compared to the arginine provision by the basal diet), or zeolite (Ze1–3: 0.125 g, 0.25 g, and 0.375 g/kg body weight/day). Lin.: Linear; Quadr.: Quadratic.

**Table 2:** Body weight (kg), feed intake (g DM/kg body weight/day) during the collection periods, urine volume (mL/kg body weight/day) and pH, in addition to amount and DM of the feces of cats fed a diet without or with arginine, ornithine, or zeolite supplementation ^1^. *n* = 10/treatment, mean and pooled SEM.

	W/O	Arginine			Ornithine			Zeolite			SEM	Polynomial Contrasts (*p* Value)					
												Arginine		Ornithine		Zeolite	
		Arg1	Arg2	Arg3	Orn1	Orn2	Orn3	Ze1	Ze2	Ze3		Lin.	Quadr.	Lin.	Quadr.	Lin.	Quadr.
Body weight	4.63	4.50	4.51	4.53	4.60	4.67	4.65	4.65	4.58	4.60	0.11	0.249	0.833	0.264	0.599	0.298	0.939
Feed intake	11.6	11.1	11.1	11.1	11.3	11.3	11.5	11.6	10.9	11.2	0.20	0.226	0.221	0.617	0.363	0.080	0.819
Urine volume	28.3	25.1	25.0	23.4	28.4	28.4	25.9	27.6	26.3	25.4	0.59	0.182	0.299	0.300	0.976	**0.029**	0.976
pH (7.00 h)	7.38	7.44	7.41	7.38	7.35	7.27	7.20	7.30	7.31	7.32	0.04	0.200	0.891	0.534	0.566	0.914	0.520
Amount of feces (g/kg BW/day)	7.83	7.51	7.14	7.46	7.71	6.31	7.58	7.78	7.16	7.73	0.31	0.540	0.273	**0.026**	0.315	0.601	0.735
Fecal DM	26.3	25.2	25.9	25.1	25.7	24.4	24.4	24.9	26.9	29.3	0.48	0.374	0.783	0.129	0.810	**0.039**	**0.029**

^1^ Basal diet supplemented without (W/O) or with arginine (Arg1–3: +50%, +75%, and +100% compared to the arginine provision by the basal diet), ornithine (Orn1–3: +100%, +150%, and +200% compared to the arginine provision by the basal diet), or zeolite (Ze1–3: 0.125 g, 0.25 g, and 0.375 g/kg body weight/day). DM: dry matter.

**Table 3:** Urinary concentration (con.) (mg/L) and excretion (excr.) (mg/kg body weight/day) of urea, ammonia, creatinine, sulfate, and hippuric acid in cats fed a diet without or with arginine, ornithine, or zeolite supplementation ^1^. *n* = 10/treatment, mean and pooled SEM.

	W/O	Arginine			Ornithine			Zeolite			SEM	Polynomial Contrasts (*p* Value)					
												Arginine		Ornithine		Zeolite	
		Arg1	Arg2	Arg3	Orn1	Orn2	Orn3	Ze1	Ze2	Ze3		Lin.	Quadr.	Lin.	Quadr.	Lin.	Quadr.
Urea con.	79.5	76.1	74.6	72.3	78.6	73.8	77.9	78.6	76.4	75.2	1.46	0.086	0.152	0.521	0.197	0.364	0.963
Urea excr.	2.28	1.88	1.86	1.73	2.23	2.08	2.01	2.18	1.99	1.93	0.06	**0.039**	**0.047**	0.188	0.303	0.094	0.918
Ammonium con.	1138	1417	1252	1228	1143	1073	1107	1021	1008	1013	34.8	0.383	0.316	0.988	0.696	0.206	0.326
Ammonium excr.	31.9	34.7	31.5	29.8	32.5	30.1	28.9	28.2	25.8	25.9	1.04	0.792	0.676	0.237	0.846	**0.038**	0.401
Creatinine con.	1175	1286	1383	1384	1249	1229	1211	1271	1443	1294	33.4	**0.005**	0.597	0.668	0.469	0.080	0.429
Creatinine excr.	33.0	30.4	33.0	31.9	35.0	35.2	31.2	34.7	33.6	32.9	0.76	0.649	0.133	0.472	0.506	0.741	0.280
Sulfate con.	2021	2021	2129	2171	2117	2097	2030	2085	2132	2056	40.1	0.059	0.665	0.606	0.400	0.610	0.439
Sulfate excr.	57.1	50.1	53.4	51.1	60.7	58.7	54.0	57.8	55.5	52.0	1.64	0.693	**0.043**	0.539	0.632	0.053	0.481
Hippuric acid con.	644	730	775	799	776	767	687	754	769	692	18.6	**0.003**	0.566	0.350	**0.038**	0.439	0.140
Hippuric acid excr.	18.0	17.5	18.9	18.3	21.9	21.5	17.7	20.7	19.0	17.6	0.53	0.335	0.436	0.656	0.054	0.356	0.065

^1^ Basal diet supplemented without (W/O) or with arginine (Arg1–3: +50%, +75%, and +100% compared to the arginine provision by the basal diet), ornithine (Orn1–3: +100%, +150%, and +200% compared to the arginine provision by the basal diet), or zeolite (Ze1).

**Table 4:** Concentrations of ammonium (µmol/g), biogenic amines (µmol/g), phenols, and indols ^1^ (µg/mg) in the feces of cats fed a diet without or with arginine, ornithine, or zeolite supplementation ^2^. *n* = 10/treatment, mean and pooled SEM.

	W/O	Arginine			Ornithine			Zeolite			SEM	Polynomial Contrasts (*P* value)					
												Arginine		Ornithine		Zeolite	
		Arg1	Arg2	Arg3	Orn1	Orn2	Orn3	Ze1	Ze2	Ze3		Lin.	Quadr.	Lin.	Quadr.	Lin.	Quadr.
Ammonium	102	93.4	81.6	103	91.4	89.7	85.9	92.5	93.3	93.8	3.03	0.860	0.084	**0.014**	0.775	0.529	0.571
Putrescine	1.67	1.94	2.01	1.54	1.46	1.48	1.19	1.13	0.96	0.82	0.09	0.663	0.141	0.128	0.887	**0.002**	0.313
Histamine	0.24	0.26	0.27	0.21	0.23	0.25	0.22	0.26	0.25	0.22	0.01	0.442	0.231	0.760	0.639	0.390	0.285
Cadaverine	0.82	1.54	1.74	1.92	1.23	1.26	1.32	0.97	0.74	0.66	0.11	0.078	0.563	0.243	0.538	0.356	0.632
Spermidine	0.73	0.68	0.70	0.63	0.60	0.65	0.55	0.55	0.55	0.63	0.03	0.432	0.884	0.263	0.772	0.401	0.058
Tyramine	0.15	0.18	0.15	0.15	0.17	0.15	0.18	0.15	0.14	0.13	0.01	0.750	0.472	0.273	0.922	0.233	0.627
Spermine	0.04	0.03	0.04	0.09	0.02	0.03	0.03	0.03	0.05	0.04	0.01	0.440	0.365	0.278	0.328	0.590	0.991
Biogenic amines (total)	3.65	4.63	4.90	4.54	3.72	3.82	3.49	3.10	2.69	2.50	0.18	0.127	0.329	0.855	0.720	**0.006**	0.663
Phenol ^3^	0.00	0.70	0.00	0.00	3.97	0.61	0.00	1.51	1.45	2.57	0.32	0.343	0.343	0.110	0.054	0.197	0.838
P-cresol	28.7	21.9	24.5	23.8	26.2	21.2	22.5	23.9	25.4	24.5	1.50	0.570	0.487	0.288	0.723	0.412	0.740
4-ethylphenol ^4^	4.36	1.55	2.83	4.09	7.24	2.77	1.33	4.24	2.62	6.11	0.63	0.947	0.173	**0.026**	0.485	0.729	0.409
Indole	230	217	221	230	242	210	243	219	199	235	6.50	0.972	0.623	0.910	0.651	0.964	0.185
3-methylindole	105	99.0	56.8	106	63.7	48.0	65.9	121	96.5	69.4	6.92	0.598	0.098	0.051	0.056	0.091	0.440
7-methylindole ^5^	45.4	83.8	47.0	62.4	40.9	56.3	102	41.7	51.4	49.5	5.67	0.862	0.218	0.091	0.169	0.802	0.914

^1^ For 2-methylindole and 2,3-dimethylindole, all values below the detection limit (0.812 µg/mL and 0.741 µg/mL). ^2^ Basal diet supplemented without (W/O) or with arginine (Arg1–3: +50%, +75%, and +100% compared to the arginine provision by the basal diet), ornithine (Orn1–3: +100%, +150%, and +200% compared to the arginine provision by the basal diet), or zeolite (Ze1–3: 0.125 g, 0.25 g, and 0.375 g/kg body weight/day). ^3^ For the groups W/O, Arg 2, Arg 3, and Orn 3: all values below the detection limit (0.756 µg/mL). Values above the detection limit: for the groups Arg1 and Orn2: n = 1; for the group Orn1: n = 4; for the groups Ze1–Ze3: n = 2. ^4^ Values above the detection limit (0.515 µg/mL): For the groups W/O, Arg2, Orn1, Ze1 and Ze3: n = 5; for the groups Arg3, Orn2 and Ze2: n = 4; for the group Arg1: n = 3; for the group Orn3: n = 2. ^5^ Values above the detection limit (0.720 µg/mL): for the groups W/O, Ze1, and Ze2: n = 9; for the groups Arg1–Arg3, Orn2, and Orn3: n = 10; for the group Orn1: n = 8; for the group Ze3: n = 7.

**Table 5:** Fecal concentration (mg/g DM) and excretion (mg/kg BW/day) as well as apparent digestibility (AD) of crude protein (%) in cats fed a diet without or with arginine, ornithine, or zeolite supplementation ^1^. *n* = 10/treatment, mean and pooled SEM.

	W/O	Arginine			Ornithine			Zeolite			SEM	Polynomial Contrasts (*P* value)					
												Arginine		Ornithine		Zeolite	
		Arg1	Arg2	Arg3	Orn1	Orn2	Orn3	Ze1	Ze2	Ze3		Lin.	Quadr.	Lin.	Quadr.	Lin.	Quadr.
Crude protein concentration	498	491	495	509	494	496	506	471	465	454	3.16	0.217	**0.025**	0.267	0.057	**0.001**	0.061
Crude protein excretion	994	873	881	893	901	729	920	873	858	949	27.7	0.286	0.095	**0.026**	**0.049**	0.498	0.212
AD crude protein	87.6	89.1	89.3	89.2	88.6	90.6	88.5	89.0	89.1	88.2	0.28	0.191	0.159	**0.035**	0.065	0.499	0.206

^1^ Basal diet supplemented without (W/O) or with arginine (Arg1–3: +50%, +75%, and +100% compared to the arginine provision by the basal diet), ornithine (Orn1–3: +100%, +150%, and +200% compared to the arginine provision by the basal diet), or zeolite (Ze1–3: 0.125 g, 0.25 g, and 0.375 g/kg body weight/day).

**Table 6:** Crude nutrient and mineral concentrations of the basal diet^1^.

Parameter	Unit	Analyzed
Dry matter	g/kg	189
Crude protein	g/kg DM	603
Crude fat	g/kg DM	251
Crude fiber	g/kg DM	8.42
Crude ash	g/kg DM	84.0
Ca	g/kg DM	15.6
P	g/kg DM	9.68
Na	g/kg DM	9.85
K	g/kg DM	9.31
Mg	g/kg DM	0.62

^1^ Composition (according to the manufacturer): meat and animal by-products, minerals, plant by-products (among others 0.4% inulin).

**Table 7:** Amino acid concentrations of the basal diet.

Amino acid	In g/kg DM
Arginine	30.2
Aspartic acid	41.8
Threonine	23.2
Serine	24.7
Glutamic acid	43.3
Glycine	36.4
Alanine	31.1
Valine	26.5
Isoleucine	18.9
Leucine	39.6
Tyrosine	17.2
Phenylalanine	24.3
Histidine	15.3
Lysine	35.4
Proline	25.9
Methionine	13.9
Cystine	6.08
Hydroxyproline	8.86
